# Hyperphosphatemia is associated with high mortality in severe burns

**DOI:** 10.1371/journal.pone.0190978

**Published:** 2018-01-09

**Authors:** George Kuo, Cheng-Chia Lee, Shih-Yi Yang, Yen-Chang Hsiao, Shiow-Shuh Chuang, Su-Wei Chang, Kun-Hua Tu, Pei-Chun Fan, Ya-Chung Tian, Yung-Chang Chen, Chih-Hsiang Chang

**Affiliations:** 1 Kidney Research Center, Department of Nephrology, Change Gung Memorial Hospital, Linkou branch, Taoyuan, Taiwan; 2 Linkou Burn Center, Department of Plastic Surgery, Chang Gung Memorial Hospital, Taipei, Taiwan; 3 Clinical Informatics and Medical Statistics Research Center, College of Medicine, Chang Gung University, Taoyuan, Taiwan; 4 Division of Allergy, Asthma, and Rheumatology, Department of Pediatrics, Chang Gung Memorial Hospital, Taoyuan, Taiwan; 5 Graduate Institute of Clinical Medicine Science, College of Medicine, Chang Gung University, Taoyuan, Taiwan; 6 Department of Nephrology, Chang Gung Memorial Hospital, Keelung Branch, Keelung, Taiwan; National Yang-Ming University, TAIWAN

## Abstract

**Introduction:**

Phosphate level is often deranged during acute illness, regardless of the presence of kidney injury or not. A few studies described that hypophosphatemia may associated with outcome in patients admitted to the burn unit, but the literatures for hyperphosphatemia is limited. Our study aims to evaluate if hyperphosphatemia, one of the sign of severe tissue damage or kidney injury, will associate with mortality of patients with severe burns.

**Materials and methods:**

The study was a post hoc analysis of prospectively collected data from patients admitted to the burn unit between September 2006 and December 2011. Patients were stratified into normophosphatemic or hyperphosphatemic group by baseline plasma phosphate level. The primary endpoint is 90-day mortality.

**Results:**

Total 301 patients were included (hyperphosphatemia: n = 52; normophosphatemia: n = 249). The hyperphosphatemic group had lower Glasgow Coma Scale, mean arterial blood pressure, hemoglobin level, albumin, and higher TBSA of burns, APACHE II score, ABSI score, Acute kidney injury (AKI), and creatinine. The 90-day mortality was higher in the hyperphosphatemic group than in the normal group (53.8% vs 18.1%, *P* < .001) and this difference was still significant when adjusting for several confounding factors (hazard ratio, 2.05; 95% CI, 1.17–3.59). Multivariable Cox analysis showed risk factors of mortality included TBSA of burns, hyperphosphatemia, reduced urine output, and APACHE II score.

**Conclusions:**

Our study found in addition to TBSA of burns and inhalation injury, baseline hyperphosphatemia in patients with severe burns is also associated with higher mortality.

## Introduction

In recent decades, several scoring systems to predict outcomes in burn patients have been evaluated. The most widely accepted risk factors for mortality in burn patients include the total body surface area (TBSA) of burns, the TBSA of full-thickness burns, age, and presence of inhalation injury.[1–13] Previous studies have also indicated the association between acute kidney injury (AKI) and mortality in burn patients.[14–16] Our previous study revealed the moderate predictive ability of AKI in burn patients.[17]

During acute illness, phosphate level is often deranged regardless of the presence of kidney injury.[18] Hypophosphatemia has been shown to be associated with patient outcome in several studies involving critically ill patients in both surgical and medical intensive care unit (ICU).[19, 20] Among patients undergoing hepatectomy, decreased phosphate level is a predictor of better survival and liver function recovery.[21–23] The results of previous studies with respect to hypophosphatemia in predicting outcomes of burn patients are also controversial.[24–26] However, hyperphosphatemia is less frequently addressed. Studies have indicated that hyperphosphatemia is associated with increased mortality among patients visiting the emergency department who were admitted for pneumonia.[27, 28] Only one case report described late-onset hyperphosphatemia in a patient with severe burns.[25]

In this study, we evaluated whether hyperphosphatemia and the trend of plasma phosphate levels are associated with the outcome of patients with severe burns.

## Materials and methods

This study was a post hoc analysis of prospectively collected data from the burn unit of a tertiary medical center in Northern Taiwan. The study protocol was approved by the Institutional Review Board of the study hospital (Chang-Gung Memorial Hospital, Taipei, Taiwan, approval number: 201701177B0). The need for informed consent was waived because of the lack of different interventions to patient groups and because patient privacy was not breached.

Patients admitted to the burn unit between September 2006 and December 2011 were enrolled. Patients aged younger than 20 years, on chronic dialysis, or with a history of organ transplant were excluded. All patients were treated by plastic surgeons and nursing staff with expertise in managing burn injuries. Patients admitted to the burn unit are initially resuscitated with lactate Ringer solution. The volume of lactate Ringer solution within the first 24 hours after admission was determined according to the Parkland formula: volume = 4 × body weight (kg) × TBSA of burn (%) × 100. Hyperphosphatemia was defined as serum phosphate level exceeding 4.5 mg/dl.[28] Urine volume was recorded. AKI was defined by criteria proposed by acute kidney injury network (AKIN). [29] We separated patients into groups based on the serum phosphate level on admission day. The 90-day mortality was the study endpoint. Patients’ demographic, burn injury, and laboratory data; Acute Physiology and Chronic Health Evaluation (APACHE) II score; and mortality were obtained from their electronic medical records.

### Statistical analysis

Continuous variables were expressed as mean ± standard deviation and compared between the study groups (normal phosphate vs hyperphosphatemia) using the Student’s *t* test. Categorical variables were expressed as frequencies with proportions and compared using Fisher’s exact test. We evaluated the change of serum phosphate from the baseline to the third day using a paired sample *t* test. To investigate whether hyperphosphatemia was associated with the risk of 90-day mortality, we established a series of multivariable Cox proportional hazard models in which the study group was treated as the explanatory variable and various covariates were introduced into the models sequentially. The survival curves of 90-day mortality were depicted using Kaplan–Meier estimates and compared between study groups using the log-rank test. Finally, we categorized the trends of plasma phosphate level into 4 groups and compared the risk of 90-day mortality among groups using the log-rank test with multiple comparisons using Bonferroni adjustment. Data analyses were conducted using SPSS 22 (IBM SPSS, Armonk, NY).

## Results

A total of 301 patients were included in this study. 3.65% of our cohort was transferred from other hospital. Table 1 shows the characteristics of patients with normal phosphate level or hyperphosphatemia. Compared with the patients with normal phosphate level, those with hyperphosphatemia had lower Glasgow Coma Scale, mean arterial blood pressure, hemoglobin level, albumin; and higher APACHE II score, ABSI score, plasma alanine aminotransferase (ALT), creatinine, blood sugar and potassium levels. The hyperphosphatemic group had less urine output on the admission day and more AKI. The 90-day mortality was also higher in the hyperphosphatemic group than in the normal group (53.8% vs 18.1%, *P* < .001). No differences were observed in terms of the proportions of preadmission co-morbidities, including diabetes, hypertension, and chronic hepatitis. The proportion of patients undergoing renal replacement therapy was not significantly different between the 2 groups (15.4% vs 8.0%, *P* = .114) (Table 1).

**Table 1:** Baseline characteristics of the patients according to serum phosphate.

Characteristics	Normal P: 2.5 to 4.5mg/dL (*n* = 249)	High P: > 4.5 mg/dL (*n* = 52)	*P*‡
Age, year	45.0±15.4	46.6±19.5	0.517
Male gender, n (%)	196 (78.7)	40 (76.9)	0.853
Diabetes mellitus, n (%)	28 (11.2)	6 (11.5)	1.000
Hypertension, n (%)	38 (15.3)	13 (25.0)	0.104
Hepatitis B/C carrier, n (%)	10 (4.0)	5 (9.6)	0.150
AKI, n (%)	14 (5.6)	20 (38.5)	<0.001
Coma scales	13.9±2.3	12.1±3.9	<0.001
Mean arterial pressure, mmHg	97.9±24.3	88.7±30.3	0.018
Urine output on the admission day, L/day	4.8±2.2	3.1±2.0	<0.001
APACHE II	9.3±6.0	16.8±8.7	<0.001
ABSI score	8.5±2.5	10.1±3.3	<0.001
Lab data			
Leukocyte count, 1000/ml	16.0±8.9	17.8±10.6	0.215
Hemoglobin, g/dl	14.7±3.2	11.9±4.5	<0.001
Platelet count, 1000/ml	194.5±74.7	177.8±92.5	0.160
ALT, u/l	51.1±72.2	114.8±174.6	<0.001
Albumin, mg/dl	2.6±0.8	2.3±0.9	0.015
Creatinine, mg/dl	0.9±0.6	1.8±1.8	<0.001
K, mmol/L	3.9±0.5	4.4±1.0	<0.001
Ca, mg/dL	8.6±0.8	8.7±0.9	0.124
P, mg/dL	3.3±0.5	6.2±1.9	<0.001
Sugar, mg/dl	162.7±56.0	196.3±94.4	0.001
Outcomes, n (%)			
Ninety-day mortality	45 (18.1)	28 (53.8)	<0.001
Renal replacement therapy	20 (8.0)	8 (15.4)	0.114
Sepsis	76 (30.5)	14 (26.9)	0.739

Abbreviations: AKI, Acute kidney injury; APACHE, Acute Physiology and Chronic Health Evaluation; ABSI, Abbreviated Burn Severity Index; ALT, alanine aminotransferase

^‡^ Comparison was made using two sample t-test for continuous data or Fisher’s exact test for categorical variables.

**Fig 1:**
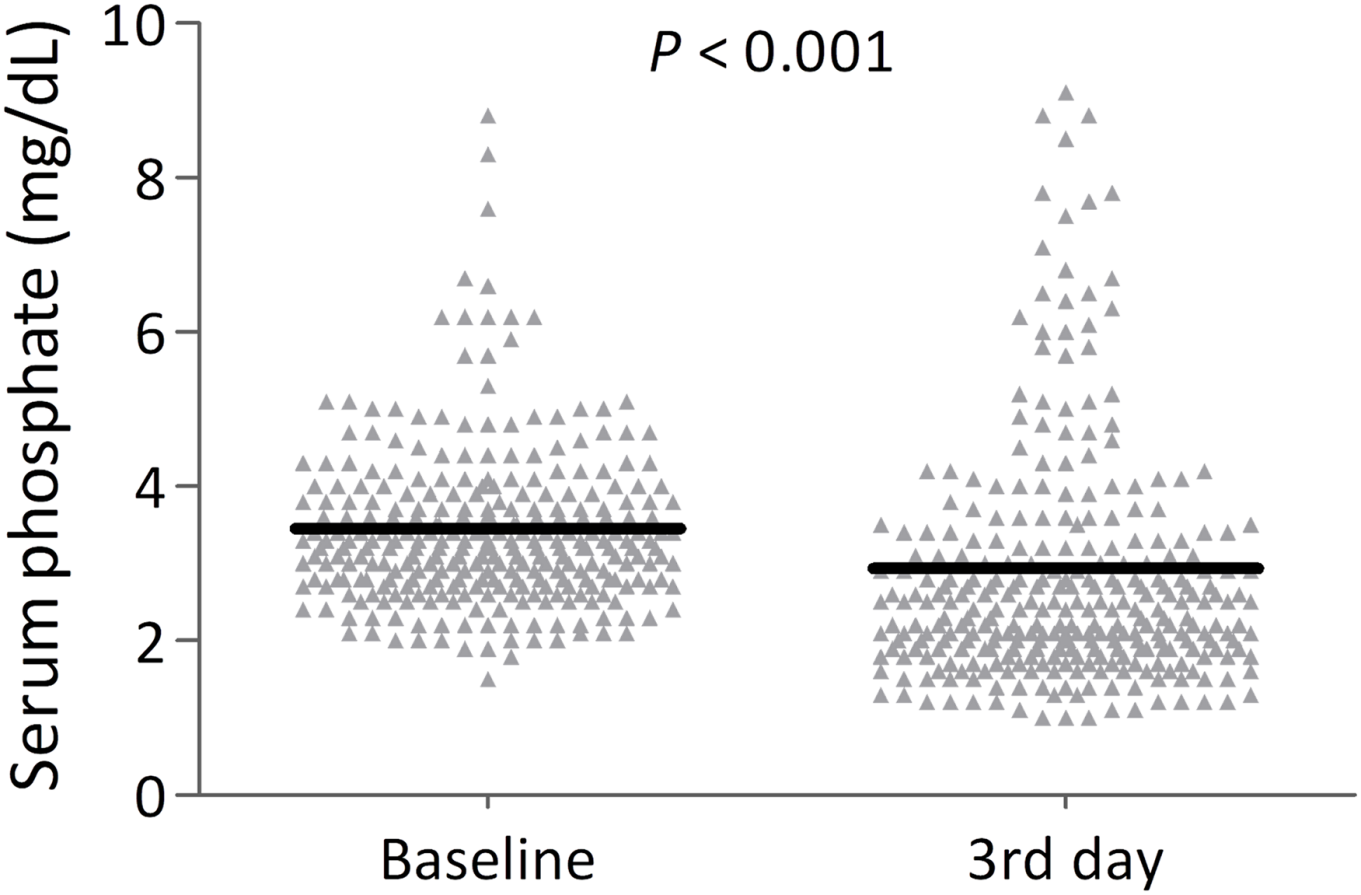
Individual values of serum phosphate levels at baseline and the third day. (Comparison was made using a paired sample *t* test).

Table 2 shows the characteristics of burn injuries between the 2 groups. Compared with patients with normal phosphate level, the TBSA of burns was larger and the proportion of flame burn was higher in patients with hyperphosphatemia. Among patients who survived longer than 3 days, the individual serum phosphate levels significantly decreased from baseline to the third day (Fig 1, *P* < .001). In addition, plasma phosphate levels were not significantly different in day 1 and day 3 in patients surviving longer than 3 days but died within 90 days (S1 Fig).

**Table 2:** Burning characteristics of the patients according to serum phosphate.

Characteristics	Normal P: 2.5 to 4.5mg/dL (n = 249)	High P: > 4.5 mg/dL (n = 52)	*P*‡
Surface area %	43.4±22.9	57.0±30.7	<0.001
Inhalation injury, n (%)	88 (35.3)	26 (50.0)	0.059
Etiologies			0.006
Flame, n (%)	163 (65.5)	42 (80.8)	
Scald, n (%)	45 (18.1)	2 (3.8)	
Electric, n (%)	14 (5.6)	2 (3.8)	
Chemical, n (%)	14 (5.6)	0 (0.0)	
Others, n (%)	13 (5.2)	6 (11.5)	

^‡^ Comparison was made using t-test for continuous data or Fisher’s exact test for categorical variable.

**Fig 2:**
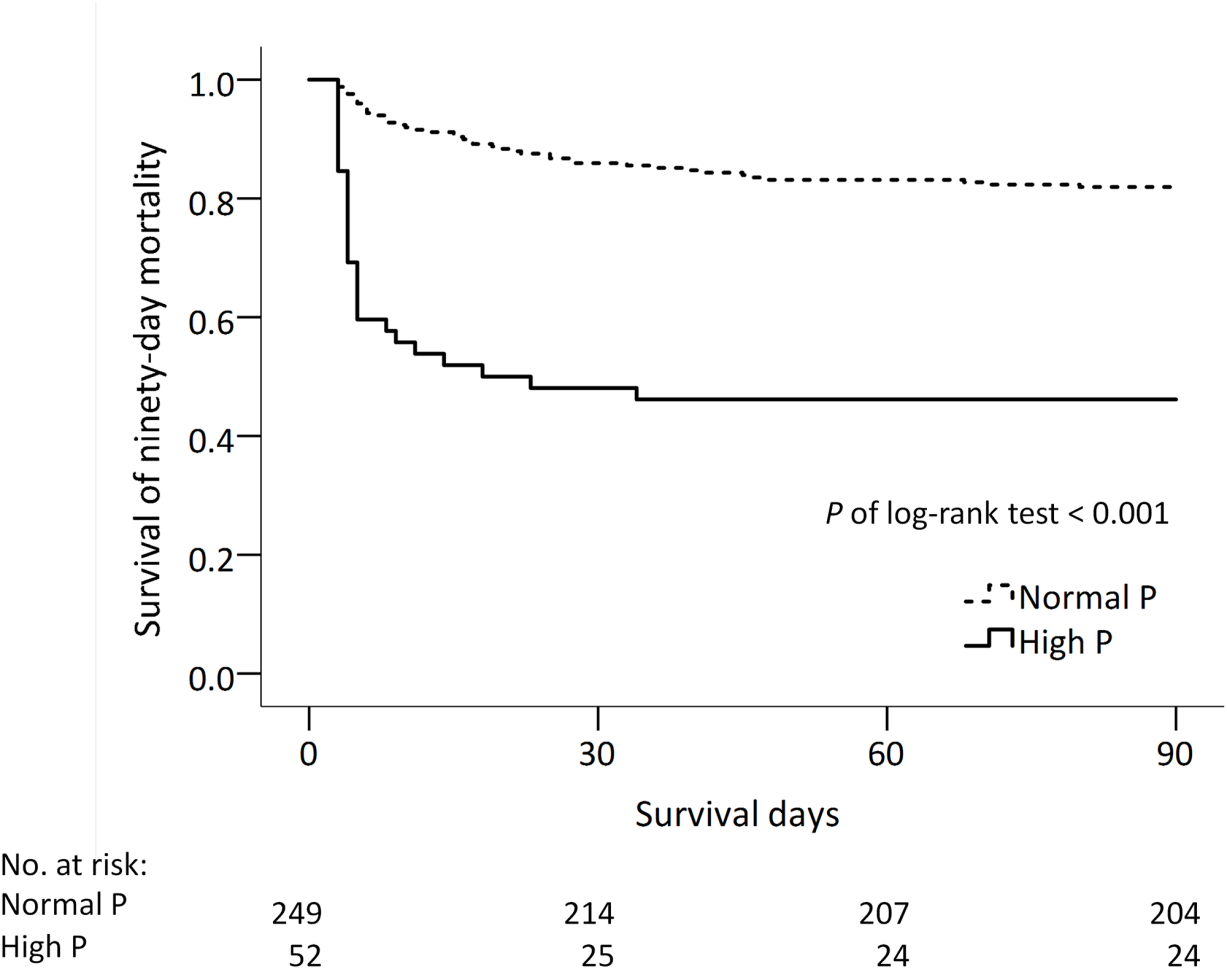
Kaplan–Meier survival curves of 90-day mortality in burn patients stratified by serum phosphate levels.

**Fig 3:**
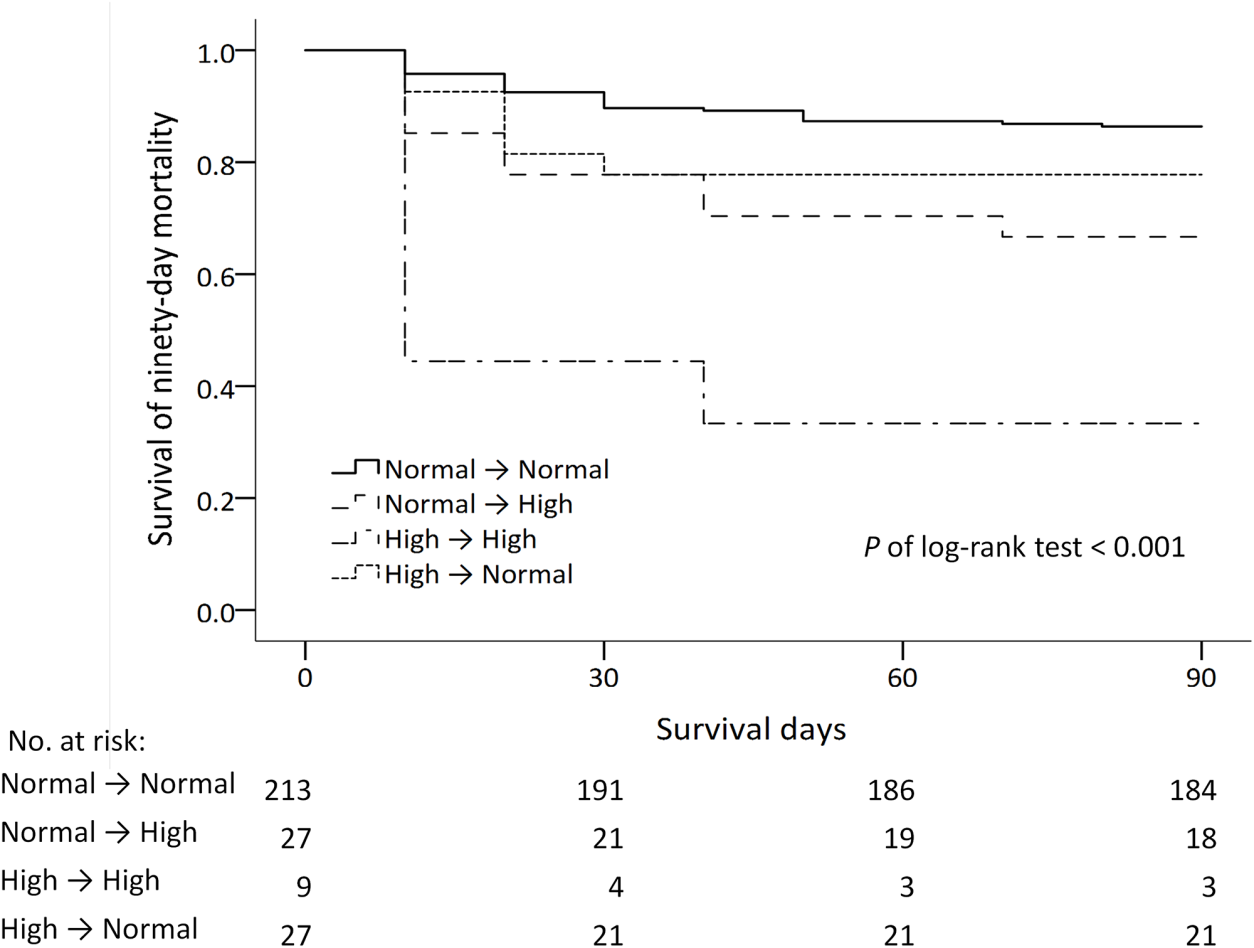
Kaplan–Meier survival curves of 90-day mortality in patients surviving more than 3 days based on different trends of plasma phosphate level. The survival rate was lower in the High → High group versus the Normal → Normal group (*P* < 0.001); the survival rates were lower in the Normal → High group and High → Normal group than in the Normal → Normal group (*P* = 0.003; *P* = 0.035). Other comparisons were not significant (*P* > 0.0083).

Patients with hyperphosphatemia had lower 90-day survival (Fig 2, log-rank test, *P* < .001). Among patients surviving longer than 3 days, the best survival was observed in patients with normal phosphate level at baseline and on the third day and the worst survival was observed in patients with persistently high phosphate level (Fig 3, log-rank test, *P* < .001). It’s noted that the survival rate was higher in the persistently normal group than in other three groups. Other comparisons among groups were not significant (*P* > .0083).

Cox analysis revealed that hyperphosphatemia was associated with a higher risk of 90-day mortality in both the unadjusted model and various adjusted models (Table 3). The result suggested that the presence of hyperphosphatemia would increase the risk of mortality independent of age, gender, surface area, inhalation injury, creatinine, urine output, AKI (model 4), APACHE II score (model 5–1) and ABSI score (model 5–2). In the final Cox model, the following covariates were found to be risk factors: TBSA of burns, hyperphosphatemia, reduced urine output, and APACHE II score (Table 4).

**Table 3:** The association of serum phosphate with risk of ninety-day mortality in various adjustment models.

Model	Presence of > 4.5 mg/dL		
	HR	95% CI of HR	*P* value
Model 1, unadjusted model	4.33	2.69–6.95	<0.001
Model 2, adjusted for age, gender	4.05	2.51–6.53	<0.001
Model 3, further adjusted for surface area, inhalation injury	3.47	2.08–5.79	<0.001
Model 4, further adjusted for creatinine, urine output, AKI	2.10	1.22–3.63	0.007
Model 5–1, based on model 4 further adjusted for APACHE II	2.05	1.17–3.59	0.013
Model 5–2, based on model 4 further adjusted for ABSI score	2.15	1.24–3.74	0.006

AKI, acute kidney injury; APACHE II, Acute Physiology and Chronic Health Evaluation; ABSI, Abbreviated Burns Severity Index; HR, hazard ratio; CI, confidence interval.

**Table 4:** Factors associated with ninety-day mortality (model 5–1 in Table 3).

Variable	HR	95% CI of HR	*P* value
Age (year)	1.01	1.00–1.03	0.192
Male gender	1.37	0.80–2.36	0.256
Surface area (per 10%)	1.05	1.04–1.07	<0.001
Inhalation injury	0.61	0.36–1.03	0.063
P > 4.5 mg/dL	2.05	1.17–3.59	0.013
Creatinine	0.94	0.73–1.21	0.615
Urine output (L/day)	0.78	0.67–0.91	0.0014
AKI	0.74	0.36–1.54	0.421
APACHE II (per unit)	1.10	1.05–1.15	<0.001

AKI, acute kidney injury; APACHE II, Acute Physiology and Chronic Health Evaluation; HR, hazard ratio; CI, confidence interval.

## Discussions

Our study demonstrated that baseline hyperphosphatemia in burn patients at the time of admission to the burn unit predicts higher mortality rate independent of the TBSA of burns, inhalation injury, and APACHE II score.

Among the scoring systems that predict the outcome in patients with burn injury,[1–3, 5, 8, 10, 30–33] %TBSA of burns, %TBSA of full-thickness burns, age, and presence of inhalation injury are the most commonly validated predictors of mortality.[13] AKI also predict higher mortality in patients with burns but has not been incorporated into most burn-specific scoring systems.[14–17, 34, 35] Yang et al found that early elevation of plasma or urine neutrophil gelatinase–associated lipocalin is associated with early AKI and mortality.[36]

Although hyperphosphatemia is commonly seen in AKI populations, it may not be fully attributed to kidney injury in our patients. These laboratory data were mostly obtained within 24 hours of the onset of burn injury because of high medical accessibility in Taiwan, and they showed that hyperphosphatemia was associated with higher TBSA of burns. The early detection of hyperphosphatemia and the association with higher TBSA of burns may reflect the severity of tissue injury and subsequent release of phosphate from the intracellular compartment.

By contrast, hypophosphatemia is commonly seen in patients during their stay in burn units and may be associated with possible complications involving multiple organs, including cardiac, respiratory, immunologic, and hematologic disorders. Lennquist et al showed that hypophosphatemia is associated with poor outcome in an early study of 33 patients with severe burns.[24] However, the association between mortality and hypophosphatemia was not seen in a later study by Tang et al involving 227 patients.[37] Despite different results regarding the impact of phosphate level on survival, these studies have all showed that a reduction in serum phosphate level occurs 2–10 days after the burn injury.[24, 37, 38] Decreases in the serum phosphate level can result from redistribution into the intracellular compartment, refeeding syndrome, gastrointestinal loss, and dilution from massive intravenous fluid resuscitation.[26, 37, 39] A protocol-based, pre-emptive phosphate supplementation has been suggested to prevent severe hypophosphatemia.[39] Among patients surviving longer than 3 days, we observed a decrease in serum phosphate level on the third day of admission, consistent with the literatures.[24, 37, 38] In addition, we also demonstrated that persistent or progressive hyperphosphatemia during early hospital days was associated with worse survival. These findings highlight that not only the baseline phosphate level but also the trend of phosphate level are associated of patient survival.

We also found that higher ABSI score was associated with poorer survival. In addition to the burn-specific scoring system, various scoring systems used in the ICU predict outcome in burn patients [4, 7, 17, 40, 41]. Although these scoring systems do not incorporate burn-specific information, they have predictive ability because they are representatives of the overall condition of critically ill patients. Survival advantage for men has been observed in some studies [9, 13, 30], but other studies have demonstrated no significant differences in mortality between men and women [3, 42–44], in line with the results of our current study.

The use of phosphate binder, adequate dialysis, and dietary phosphate restriction for controlling hyperphosphatemia has been largely used in the CKD and ESRD populations. [45] There is a lack of direct evidence of using such approaches in patients with acute, critical illness. Based on our practice and this study, we could not conclude that the use of phosphate binder or hemodialysis will improve the outcome of hyperphosphatemic patients or not. Further study, especially a randomized controlled trial, might be required to assess the effect of phosphate lowering therapy adding on the optimal burn care.

There are several limitations to our study. First, these data were collected in a single tertiary medical center, which may limit the generalization of our findings. Second, expansion our result in patients transferred from other hospital should be careful since their level of phosphate might not reflect the baseline condition after burn injury. Third, we only uniformly measure the plasma phosphate level on the first and third day of admission. Thus, we did not have adequate evidence to conclude the possible association between the temporal change of phosphate level beyond the third hospitalization day and patient outcome. Finally, we did not investigate whether the measurement of serum phosphate improves the predictive ability of the burn-specific scoring system. Further studies are required to validate our findings within a larger population and to estimate any additional improvement of these findings into current predictive scoring systems.

## Conclusions

Our results showed that baseline hyperphosphatemia and persistent or progressive hyperphosphatemia are associated with higher mortality in burn patients, independent of traditional risk factors such as the TBSA of burns and inhalation injury.

## Supporting information

S1 Fig Individual values of serum phosphate at baseline and the third day for the 51 patients who survived longer than 3 days but died within 90 days. (Comparison was made using paired sample t-test).
(TIF)
