# Supplementary figures and images for: Hyperphosphatemia is associated with high mortality in severe burns
Source: PLoS One. 2018 Jan 9;13(1):e0190978. doi: 10.1371/journal.pone.0190978 (PMC5760089; doi:10.1371/journal.pone.0190978)

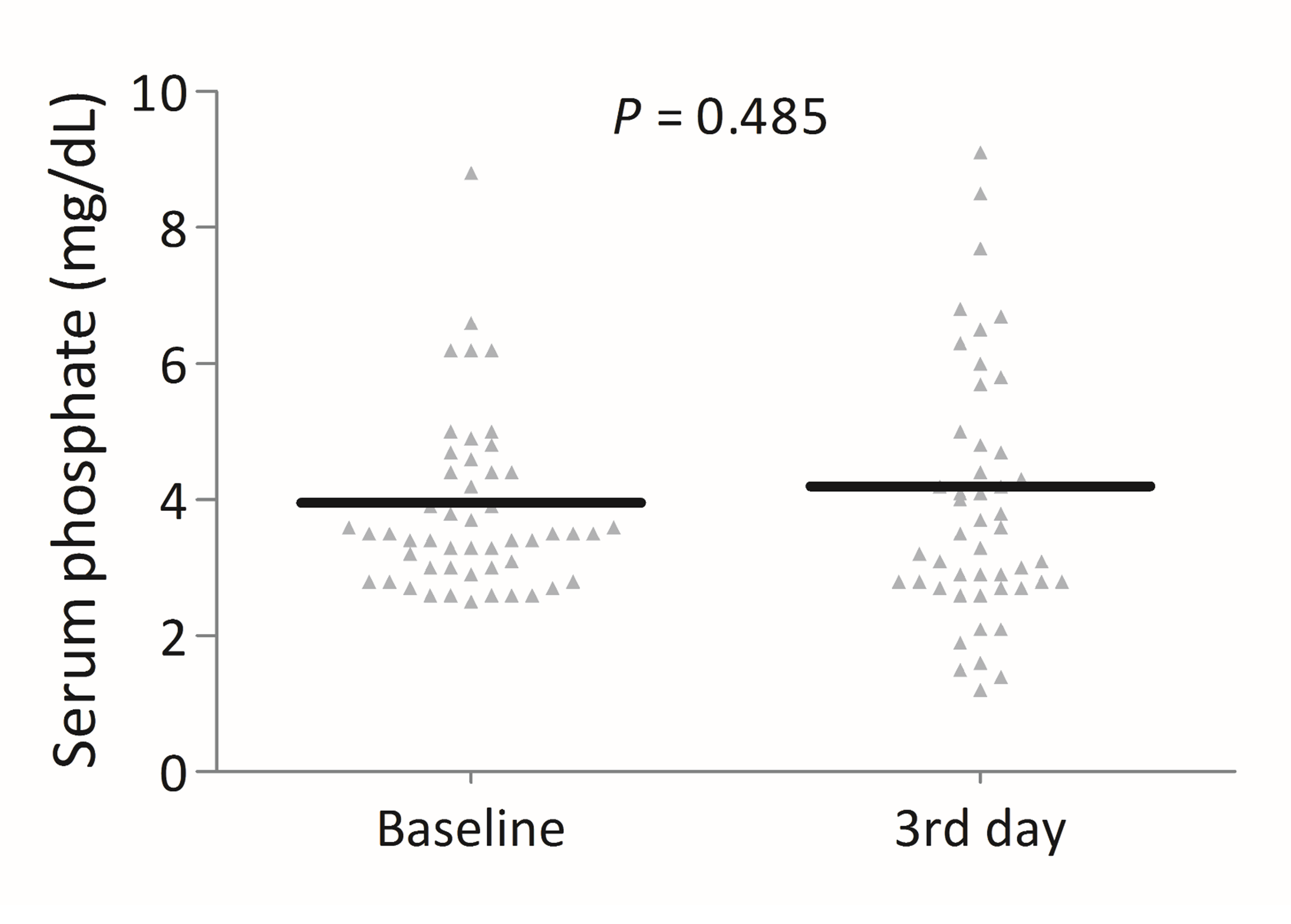

Supplement: S1 Fig — (Comparison was made using paired sample t-test). (TIF) [file pone.0190978.s001.tif]
